# *Nocardia* Infection in Nephrotic Syndrome Patients: Three Case Studies and A Systematic Literature Review

**DOI:** 10.3389/fcimb.2021.789754

**Published:** 2022-01-24

**Authors:** Yan Cheng, Tian-yi Wang, Hong-li Yuan, Wei Li, Jing-ping Shen, Zheng-xin He, Jing Chen, Jie-ying Gao, Fu-kun Wang, Jiang Gu

**Affiliations:** ^1^ Department of Basic Medical Laboratory, The 980th Hospital of the PLA Joint Logistical Support Force (Bethune International Peace Hospital), Shijiazhuang, China; ^2^ Department of Respiratory Medicine, The 980th Hospital of the PLA Joint Logistical Support Force (Bethune International Peace Hospital), Shijiazhuang, China; ^3^ Department of Radiology, The 980th Hospital of the PLA Joint Logistical Support Force (Bethune International Peace Hospital), Shijiazhuang, China; ^4^ Department of Clinical Laboratory, The 980th Hospital of the PLA Joint Logistical Support Force (Bethune International Peace Hospital), Shijiazhuang, China; ^5^ Department of Nutrition, Beidaihe Rehabilitation and Recuperation Center, Qinhuangdao, China; ^6^ Department of Microbiology and Biochemical Pharmacy, College of Pharmacy, Army Medical University, Chongqing, China

**Keywords:** *Nocardia*, nocardiosis, nephrotic syndrome, immunosuppression, infection

## Abstract

**Objective:**

The multicenter literature review and case studies of 3 patients were undertaken to provide an updated understanding of nocardiosis, an opportunistic bacterial infection affecting immunosuppressed nephrotic syndrome (NS) patients receiving long-term glucocorticoid and immunosuppressant treatment. The results provided clinical and microbiological data to assist physicians in managing nocardiosis patients.

**Methods:**

Three cases between 2017 and 2018 from a single center were reported. Additionally, a systematic review of multicenter cases described in the NCBI PubMed, Web of Science, and Embase in English between January 1, 2001 and May 10, 2021 was conducted.

**Results:**

This study described three cases of *Nocardia* infection in NS patients. The systematic literature review identified 24 cases with sufficient individual patient data. A total of 27 cases extracted from the literature review showed that most patients were > 50 years of age and 70.4% were male. Furthermore, the glucocorticoid or corticosteroid mean dose was 30.9 ± 13.7 mg per day. The average time between hormone therapy and *Nocardia* infection was 8.5 ± 9.7 months. Pulmonary (85.2%) and skin (44.4%) infections were the most common manifestations in NS patients, with disseminated infections in 77.8% of patients. Nodule/masses and consolidations were the major radiological manifestations. Most patients showed elevated inflammatory biomarkers levels, including white blood cell counts, neutrophils percentage, and C-reactive protein. Twenty-five patients received trimethoprim-sulfamethoxazole monotherapy (18.5%) or trimethoprim-sulfamethoxazole-based multidrug therapy (74.1%), and the remaining two patients (7.4%) received biapenem monotherapy. All patients, except the two who were lost to follow-up, survived without relapse after antibiotic therapy.

**Conclusions:**

Nephrotic syndrome patients are at high risk of *Nocardia* infection even if receiving low-dose glucocorticoid during the maintenance therapy. The most common manifestations of nocardiosis in NS patients include abnormal lungs revealing nodules and consolidations, skin and subcutaneous abscesses. The NS patients have a high rate of disseminated and cutaneous infections but a low mortality rate. Accurate and prompt microbiological diagnosis is critical for early treatment, besides the combination of appropriate antibiotic therapy and surgical drainage when needed for an improved prognosis.

## Introduction

Nocardia species are aerobic, Gram-positive, filamentous, beaded, weakly acid-fast branching bacilli found worldwide in soil and water (Brown-Elliott et al., 2006; Wilson, 2012). More than 100 different *Nocardia* species have been identified by phenotypic identifications, molecular methods and 16S rRNA gene sequencing (https://www.bacterio.net) up to now, and over 50 of them have been reported pathogenic to humans (Conville et al., 2018).

*Nocardia*, an opportunistic pathogen, infects humans *via* respiratory inhalation and injured skin. The organism causes pulmonary, superficial cutaneous, and subcutaneous infections, and can spread through the blood causing disseminated infection (Beaman and Beaman, 1994; Wang et al., 2015). Chronic lung disease and immunosuppression caused by glucocorticoids or other immunosuppressive therapies, human immunodeficiency virus (HIV) infection, solid organ transplantation, and chemotherapy for neoplasm are the common risk factors for *Nocardia* infections (Conville et al., 2003; Liu et al., 2011; Molina et al., 2018; Zia et al., 2019). Recent years have seen an increase in nocardiosis incidences with extensive immunosuppressive therapies.

Nephrotic syndrome (NS), caused by glomerular permeability abnormality, is a clinical syndrome with massive proteinuria responsible for hypoalbuminemia (<30 g/L), hyperlipidemia, edema, and various complications. Impaired renal function, administration of glucocorticoids, and immunosuppressant use in NS patients may lead to immune disorders, making them more susceptible to various infections (Li et al., 2017; Wang et al., 2019). NS patients with long-term glucocorticoids and (or) immunosuppressants treatments have high morbidity rates of *Nocardia* infection. *Nocardia* infection in NS patients was first described in 1962 by Kerbel NC (Kerbel, 1962). Only a few case reports of *Nocardia* infection in NS patients are available up to now, and the two latest single-center literature reviews were published in 2020 (Guo et al., 2020; Han et al., 2020). However, there are hardly any multicenter retrospective reviews. The present multicenter study reports *Nocardia* infection in 3 NS patients between 2017 and 2018 in our hospital, summarizes the available literature to understand the infectious disease, and provides clinical and microbiological data to assist physicians in managing nocardiosis patients.

## Materials and Methods

### Single-Center Case Report

Three patients with a medical history of NS and diagnosed with nocardiosis at the 980th Hospital of the PLA Joint Logistical Support Force from 2017 to 2018 were retrospectively reviewed for clinical history and characteristics, laboratory data, imaging features, microbiological data, and treatment data. The *Nocardia* infection was defined by clinical features, radiographic manifestations, pathogen identification, and post-treatment radiological or clinical condition improvements. Disseminated nocardiosis was defined as the involvement of at least two noncontiguous organs or demonstration of bloodstream infection (Margalit et al., 2021).

Different clinical specimens, including sputum, pus, pleural, and bronchoalveolar lavage, were inoculated on the blood-containing medium and Lowenstein-Jensen medium at 35°C for 3-21 days under aerobic conditions. Presumptive *Nocardia* species were identified by Gram staining, modified acid-fast staining, and acid-fast staining, and then confirmed by 16S rRNA gene sequencing. Based on Clinical and Laboratory Standards Institute (CLSI, 2018) (Woods et al., 2011) guidelines, antimicrobial susceptibility testing (AST) was performed by broth microdilution (BMD), and the minimum inhibitory concentrations (MICs) were interpreted according to the CLSI susceptibility breakpoints.

Inflammatory biomarkers, including white blood cell (WBC) counts, neutrophils percentage, C-reactive protein (CRP), procalcitonin (PCT), and erythrocyte sedimentation rate (ESR), were analyzed to evaluate the infection. Serum albumin and creatinine were measured as the renal function indicators.

### Literature Review

We searched Pubmed, Web of Science, and Embase to identify original research, case reports, case series, and review articles with detail medical history and laboratory data published between January 1, 2001 and May 10, 2021. The search keywords included “Nephrotic syndrome”, “Nocardia”, “Nocardiosis”, “steroid therapy”, “glucocorticoid therapy”, “Kidney disease”, and “infection”. All relevant, available full texts published in English were extracted.

### Statistical Analysis

Data analysis was performed using SPSS 20.0 (SPSS Inc., Chicago, IL, USA). Continuous variables were presented as the means with standard deviations (SDs). Significant differences between any two groups were tested using the χ2 test and the Fisher’s exact test when the value in any group was below 5. The *P-*value of <0.05 was considered to be statistically significant.

## Results

### Single-Center Three Case Reports

Three cases of nocardiosis with NS identified between January 1, 2017 and December 31, 2018 were studied. **Table S1** presents the antimicrobial susceptibility patterns for *Nocardia*; *N.cyriacigeorgica*, *N.brasiliensis*, and *N.farcinica*. The three cases are discussed as follows.

#### Case 1

**Figure 1:**
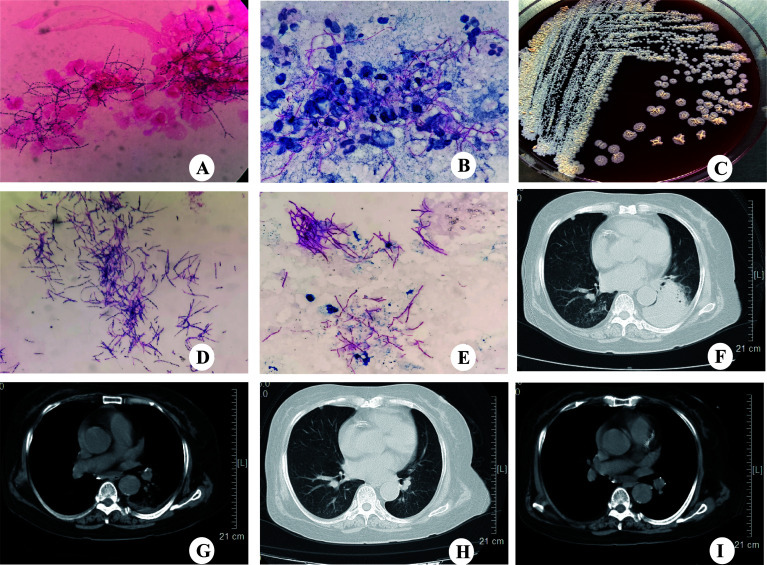
CT scanning and microbiological identification of case 1. **(A)** Gram staining of BALF showing Gram-positive, filamentous branching bacilli (magnification, ×100). **(B)** Modified acid-fast staining of BALF showing filamentous, weakly acid-fast branching bacilli (magnification,×100). **(C)** Colonies of *N.cyriacigeorgica* cultured on blood agar for 72h. **(D)** Gram staining showing the Gram-positive, filamentous branching and beaded structure of *N.cyriacigeorgica* (magnification,×100). **(E)** Modified acid-fast staining showing filamentous, weakly acid-fast branching bacilli (magnification,×100). **(F)** Chest CT showing consolidation in the left lower lobe with left-sided pleural effusion. **(G)** Chest CT showing left-sided pleural effusion. **(H)** Chest CT showing consolidation in the left lower lobe disappeared following treatment. **(I)** Chest CT showing left-sided pleural effusion disappeared following treatment.

**Figure 2:**
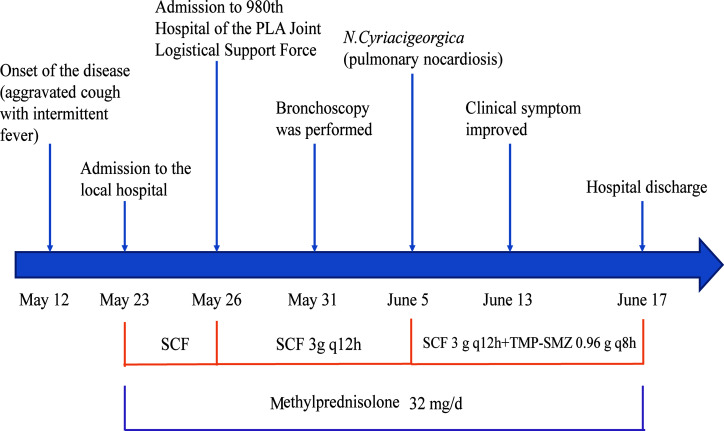
Treatment flow diagram of case 1. TMP-SMX, trimethoprim-sulfamethoxazole; SCF, cefoperazone/sulbactam.

In May 2017, a 71-year-old women was admitted to our hospital with an aggravated cough and intermittent fever for 14 days. Three days before the admission, the patient was initially treated with cefoperazone/sulbactam in a local hospital because the radiographic manifestations suspected bacterial pneumonia. However, her symptoms did not improve, and the fever was persistent; she was then transferred to our hospital for further diagnosis and treatment. She had a 3-month history of NS, and the renal biopsy confirmed focal segmental glomerulersclerosis. Subsequently, she was on steroid therapy (methylprednisolone, 32 mg/d) for 3 months. On admission, the patient’s body temperature was 36.2°C. The WBC was 15.8×10^9^/L with 89% neutrophils; ESR and CRP were elevated to 96 mm/h and 87.3 mg/L, respectively. The 24-h urine protein stood at 6687.5 mg/d, the serum albumin was 25.7 g/L, and the serum β-D-glucan was normal. Chest computed tomography (CT) scan revealed cord-like high-density shadows in both lungs and consolidation in the left lower lobe with left-sided pleural effusion (**Figure 1**). Bronchoscopy showed white purulent mucus found on the left lower bronchi (**Supplementary Figure S1**). Bronchoalveolar lavage fluid (BALF) showed branched neutrophilia, and the Gram staining and modified acid-fast staining revealed filamentous, beaded, branching bacilli (**Figure 1**). The BALF culture on the blood agar revealed *Nocardia* spp., identified as *N.cyriacigeorgica* by 16S rRNA sequencing (**Supplementary Figure S2**). Further investigations of the disseminated infection, including brain CT (**Supplementary Figure S1**) and blood culture, were negative. Pulmonary nocardiosis was diagnosed based on the clinical data, imageological characteristics, and pathogen identification. The patient was administered intravenous cefoperazone/sulbactam (3 g twice daily) and oral trimethoprim-sulfamethoxazole (TMP-SMX, 0.96 g thrice daily) for 14 days based on the bacterial species and AST results. A follow-up CT after 2 weeks showed the disappearance of left pleural effusion and flake consolidation in the left lower lobe. She was discharged on a 6-month course of oral TMP-SMX therapy after 20 days of hospitalization. **Figure 2** shows the treatment flow diagrams of the case.

#### Case 2

A 68-year-old man presented with fever and coughing up purulent sputum. He had been diagnosed with NS one year prior. Renal biopsy showed membranous nephropathy, and he was administered a daily dose of 30 mg prednisone and 100 mg cyclosporine each. One month prior to the admission, he was initially diagnosed with *Aspergillus fumigatus* pneumonia and received the oral voriconazole treatment. The 2-week antifungal therapy improved the patient’s symptoms both clinically and radiologically. He was discharged on a regimen of prednisone (30 mg/d), cyclosporine (100 mg/d), and voriconazole (400 mg/d). On admission, a physical examination revealed low fever (37.6°C). After one-year-steroid therapy, the 24-h urinary protein loss was reduced to 656 mg/d, and the serum albumin levels rose to 24.6 g/L. The WBC count was 10.7×10^9^/L with 91% neutrophils, and the CRP and PCT were elevated to 188 mg/L and 1.6 ng/mL, respectively. His fasting blood sugar was 9.2 mmol/L and the glycosylated hemoglobin was 10.6%. Laboratory investigations regarding lymphocyte count were abnormal: CD3+ 53.6%, CD3+CD4+ 12.8%, CD3+CD8+ 40.6%, CD4/CD8 0.32, indicating suppressed immunity. Serum β-D-glucan and galactomannan were normal. Chest CT revealed multiple flake and cord-like high-density shadows in both lungs near the pleura, multiple cavities in the right upper lobe, bilateral pleural effusion, and emphysema in both lungs (**Supplementary Figure S3**). Pathogens isolated from sputum were ultimately identified as *N.brasiliensis* by 16S rRNA gene sequencing (**Supplementary Figure S4**), but the blood cultures were negative. The patient was administered intravenous moxifloxacin (0.4 g/d) and oral TMP-SMX (0.96 g thrice daily) for 14 days. A follow-up chest CT revealed the reduced size of the previously detected cavities (**Supplementary Figure S3**) and improved clinical symptoms after 21-day hospitalization. Unfortunately, the patient refused further medication and was discharged on a 6-month course of oral TMP-SMX therapy but was lost to follow-up. **Supplementary Figure S5** presents the treatment flow diagrams of the case.

#### Case 3

During the autumn of 2017, a 69-year-old man suffering from edema in both lower legs was admitted to our hospital. He was diagnosed with NS (membranous nephropathy), and an intravenous methylprednisolone therapy (32 mg/d) was initiated. There was no improvement 1 month post-treatment, so the patient was started on a combination therapy of oral prednisone (25 mg/d) and hemodialysis. His renal disease stabilized, so the prednisone dosage was reduced to 15 mg/d as the maintenance therapy. After 2 months of combination therapy, the 24-h urinary protein loss was reduced to 378 mg/d, while the serum albumin level rose to 22.9 g/L. However, he developed low fever, cough, and chest pain. The laboratory examination showed elevated WBC count (11.8×10^9^/L) with 91% neutrophils, CRP (26 mg/L), PCT (2.9 ng/ml), and ESR (90 mm/h). Serum β-D-glucan and galactomannan were normal. Chest CT revealed bilateral inflammation, cavities in the right upper lobe, and multiple nodules in both lungs. *Mycobacterium tuberculosis-*specific T lymphocyte (T spot-TB) was positive, but *M tuberculosis* was not founded in the sputum. Based on the clinical features, imageological characteristics, and lab tests, the patient was administrated a combination of isoniazid and rifampin because *M tuberculosis* was suspected of etiological agent. Nevertheless, his symptoms did not improve, and the chest CT revealed enlarged nodules in the right middle lobe and aggravated inflammation in the left lower lobe. A firm abscess (2cm×1cm in diameter) which manifested as tender was founded in the left upper groin. Ultrasonography (USG) of the abscess showed thickened subcutaneous soft tissue and non-uniform internal echo (**Supplementary Figure S6**). He had no history of trauma. The abscess was drained, and the yellowish purulent discharge was sent to the laboratory for culture. The Gram-and modified acid-fast staining of the sputum showed filamentous, beaded, branching bacilli which was suspected of *Nocardia* spp. (**Supplementary Figure S6**). The sputum was resubmitted, cultured aerobically on the blood agar after digestion with 2% potassium hydroxide (KOH), and incubated at 37°C to confirm the presence of *Nocardia* spp. The pus and sputum culture presented yellowish *Nocardia* spp., identified as *N.farcinica* by gene sequencing (**Supplementary Figure S7**). Pus and sputum cultures, and morphological evidence of sputum were positive for *Nocardia* spp. and disseminated nocardiosis was diagnosed. Antibiotic therapy was replaced with intravenous cefatriaxone (2 g/d) and oral TMP-SMX (0.96 g thrice daily) for 2 weeks. The patient was discharged as his clinical condition improved after 43 days of hospitalization. He continued oral TMP-SMX monotherapy for 6 months and remained in good condition. The treatment flow diagrams of the case are presented in **Supplementary Figure S8**.

### Literature Review

We performed a systematic literature search of previous articles and identified 13 articles on nocardiosis with NS; 3 of them were excluded (1 article published in Japanese, 1 article published in Danish, 1 article published in Turkish). Ten articles described 24 cases that had sufficient individual patient data. **Table S2** summarizes these publications (Hwang et al., 2004; Sirijatuphat et al., 2013; Zhou et al., 2014; Chen et al., 2016; Zhu et al., 2017; Xu et al., 2018; Guo et al., 2020; Han et al., 2020; Sah et al., 2020; Yang et al., 2020). A total of 27 cases of nocardiosis with NS were assessed, including the three cases of nocardiosis in our hospital. Demographic characteristics, clinical features, laboratory data, radiological features, microbiological results, treatment data, and patient outcomes were collected and summarized (**Tables 1**, **2**).

**Table 1:** Epidemiographical and characteristic data of 27 nocardiosis patients with nephrotic syndrome.

Case	Authors	Age/gender	Concomitant diseases	Treatment of NS	Type of kidney disease	Duration of hormone therapy(months)	Hormone doses when infected (mg/d)	Clinical symptoms	Chest imaging	Infection sites	Antibiotic treatment	If surgical drainage	Duration of antibiotic therapy	Outcome
1		70/F		Methylprednisolone	FSGS	3	32	Fever, cough, expectoration	Pleural effusion, consolidation, bronchiectasis	Lung	TMP-SMZ+SCF	No	6 months	Survival
2		68/M	COPD, diabetes	Prednisone +CsA	MN	12	30	Fever, cough, expectoration, chills	Pleural effusion, consolidation, cavitary, nodules, emphysema, pulmonary bulla, lung abscess	Lung	TMP-SMZ+MXF	No	Loss to follow up	Loss to follow up
3		68/M	COPD, steroid diabetes	Prednisone	MN	6	15	Fever, cough, expectoration, chest pain, subcutaneous abscesses	Nodules, cavitary, emphysema, pulmonary bullae	Lung, skin	TMP-SMZ+CTR	Yes	6 months	Survival
4	(Xu et al., 2018)	58/M	Steroid diabetes	Prednisone	MN	4	20	Fever, cough, night sweat	Multiple bilateral nodule, lung and brain abscess	Lung, eye, brain, blood	TMP-SMX+IMP	No	>6 months	Survival
5	(Chen et al., 2016)	63/M	Steroid diabetes, hypertension, HBV infection	Methylprednisolone	MsPGN	9	8	Fever, chill, subcutaneous abscesses	Normal	Skin	TMP-SMX+LZD	Yes	6 months	Survival
6	(Zhu et al., 2017)	60/M		Methylprednisolone+ tacrolimus	MN	14	NS	Subcutaneous abscesses	Nodules	Lung, skin	TMP-SMX+CTR	No	6 months	Survival
7	(Sah et al., 2020)	61/M		Corticosteroid	FSGS	2	60	Fever, cough, swell of right thigh	Consolidation, pleural effusion	Lung, skin	TMP-SMX+AMK+LZD	No	6 months	Survival
8	(Zhou et al., 2014)	55/M	HIV	Prednisone+tacrolimus	MN	13	30	Fever, cough, expectoration, hard subcutaneous nodule, left hip pain	Nodule, brain abscess, pleural effusion	Lung, hip, brain	TMP-SMX	Yes	6 months	Survival
9	(Hwang et al., 2004)	60/M		Prednisone	NS	8	25	Fever, cough, expectoration	Bilateral multiple cavitary nodules	Lung	TMP-SMX	No	6 months	Survival
10	(Sirijatuphat et al., 2013)	53/M	Steroid diabetes	Prednisolone	MCN	5	40	Chest pain, dyspnea	Cardiomegaly, pericardial fluid, atelectasis, interlobular septal thickening	Pericardium, lung	TMP-SMX+IMP	Yes	12 months	Survival
11	(Guo et al., 2020)	38/F		Glucocorticiod^b^, AZA, MMF, cyclophosphamide, LEF+CsA MMF+cyclophosphamide	LN^a^	27	30	Fever, subcutaneous abscesses		Lung, skin	TMP-SMX+CTR	Yes	4-6 months	Survival
12	(Guo et al., 2020)	42/F	Steroid diabetes	Glucocorticiod^b^, MMF+tacrolimus	LN-IV	2	20	Fever, subcutaneous and perihepatic abscesses		Lung, skin, perihepatic, adrenal gland	BPM	Yes	4-6 months	Survival
13	(Guo et al., 2020)	45/M		Glucocorticiod^b^, CsA	MN	11	25	Fever, subcutaneous abscesses	Pleural effusion	Lung, skin, pleural cavity, brain	TMP-SMX+BPM	Yes	4-6 months	Survival
14	(Guo et al., 2020)	23/F		Glucocorticiod^b^, Cyclophosphamide+tacrolimus+CsA+TW	LN-V+III	6	30	Fever, subcutaneous abscesses		Lung, skin	BPM	Yes	4-6 months	Survival
15	(Guo et al., 2020)	26/F	Steroid diabetes	Glucocorticiod^b^, tacrolimus	MCN	6	30	Fever	Mass pleural effusion, brain abscess	Lung, pleural cavity, brain	TMP-SMX	No	4-6 months	Survival
16	(Guo et al., 2020)	26/M		Glucocorticiod^b^, CsA	NS^a^	6	60	Fever, subcutaneous abscesses		Lung, skin, perihepatic	TMP-SMX	Yes	4-6 months	Survival
17	(Guo et al., 2020)	47/F	Diabetes	Glucocorticiod^b^, TW+tacrolimus	MN	4	35	Fever, subcutaneous abscesses		Lung, skin	TMP-SMX+TZP	Yes	4-6 months	Survival
18	(Guo et al., 2020)	22/F		Glucocorticiod^b^, TW+tacrolimus	IgAN	51	50	Fever	Mass pleural effusion	Lung, pleural cavity	TMP-SMX+SCF	No	4-6 months	Survival
19	(Guo et al., 2020)	33/M	Steroid diabetes	Glucocorticiod^b^, tacrolimus	NS^a^	4	30	Subcutaneous abscesses		Lung, skin, eye	TMP-SMX+BPM	Yes	4-6 months	Survival
20	(Guo et al., 2020)	73/M		Glucocorticiod^b^, TW	MN	5	60	Fever	Brain abscess	Lung, brain	TMP-SMX+BPM+LZD	No	4-6 months	Survival
21	(Guo et al., 2020)	63/M	Steroid diabetes	Glucocorticiod^b^, Cyclophosphamide+CsA	MN	4	30	Fever, subcutaneous abscesses		Lung, skin	TMP-SMX+BPM	Yes	4-6 months	Survival
22	(Han et al., 2020)	50-60/M		Methylprednisolone+ tacrolimus	MN	6	16	Fever, cough, expectoration, subcutaneous abscesses	Brain abscess	Lower, abdomen, hip, brain	TMP-SMX+IPM	Yes	4.5 months	Survival
23	(Han et al., 2020)	20-30/M	Chronic HBV infection, steroid diabetes	Methylprednisolone	MN	5	16	Cough, expectoration, subcutaneous abscesses		Thigh	TMP-SMX	No	3 months	Survival
24	(Han et al., 2020)	60-70/M	Diabetes	Methylprednisolone	NS^a^	4	32	Subcutaneous abscesses		Middle finger	TMP-SMX+mezlocillin/sulbactam	No	Loss to follow up	Loss to follow up
25	(Han et al., 2020)	50-60/M		Methylprednisolone	MN	4	28	Fever, cough, expectoration, subcutaneous abscesses	Lung abscess	Lung, neck	TMP-SMX+LZD	No	3 months	Survival
26	(Han et al., 2020)	50-60/F	Steroid diabetes	Methylprednisolone+tacrolimus	NS^a^	6	20	Subcutaneous abscesses	Pleural effusion	Lung, neck, hip	TMP-SMX+LZD	No	3 months	Survival
27	(Yang et al., 2020)	65/M		Prednisone+tacrolimus	MN	3	NS	Cough, expectoration, chest pain, multiple abscesses	Pleural effusion, lung abscess	Lung, lower, limb, cheek	TMP-SMX+CTX+LEV	No	ND	Survival

F, female; M, male; ND, not describe; NS, nephrotic syndrome; COPD, chronic obstructive pulmonary disease; FSGS, Focal segmental glomerulersclerosis; MN, membranous nephropathy; MsPGN, mesangial proliferative glomerulonephritis; MCN, minimal change nephrosis; LN, lupus nephritis; IgAN, immunoglobulin A nephropathy; AZA, azathioprine; MMF, mycophenolate mofetil; LEF, leflunomide; CsA, cyclosporine; TW, tripterygiumwifordii; CTR, cefatriaxone; CTX, cefotaxime; TMP-SMX,trimethoprim-sulfamethoxazole; BMP, biapenam; TZP, piperacillin/tazobactam; SCF, cefoperazone/sulbactam; LZD, linezolid; AMK, amikacin; IPM, imipenem; MXF, Moxifloxacin; LEV, levofloxacin.

a Without pathological diagnosis.

b not describe type of glucocorticoid.

**Table 2:** Laboratory examination results of 27 nocardiosis patients with nephrotic syndrome.

Case number	WBC (×10^9^/L)/neutrophils percentage (%)	CRP (mg/L)	PCT (ng/mL)	Alb (g/L)	SCr (umol/L)	Hb (g/L)	CD4^+^ lymphocyte	Identification method	Specimen type	Nocardia species	AST
1	15.8/89	87.3	1.1	25.7	99	109	ND	16S rRNA	BALF	N.cyriacigeorgica	Yes
2	10.7/91	188	1.6	24.6	81	96	12.8%	16S rRNA	Sputum	N.brasiliensis	Yes
3	16.9/91	26	2.9	22.9	583	109	ND	16S rRNA	Sputum, pus	N.farcinica	Yes
4	11.5/75.8	59.9	ND	ND	ND	ND	ND	Culture	Sputum, blood	ND	Yes
5	17.5/88.4	130	0.3	ND	ND	ND	45%	16S rRNA	Pus	N.brasiliensis	Yes
6	8.4/85.5	12.2	ND	18.4	131	ND	28.6%	16S rRNA	Pus	N.farcinica	Yes
7	16/82	112	0.4	ND	79.6	ND	ND	MALDI-TOF MS	Sputum, pus	N.otitidiscaviarum	Yes
8	19.7/85	ND	ND	20.2	ND	ND	ND	Culture	Sputum, pus	N.asteroides	ND
9	14.4/ND	45.7	ND	ND	ND	ND	ND	Culture	Sputum, BALF	N.asteroides	ND
10	23.4/93	ND	ND	27	ND	ND	ND	16S rRNA	Pericardial fluid	N.farcinica	Yes
11	17.2/ND	42	0.8	33.3	160	80	51/ul	Culture	Pus	N.asteroides	ND
12	26.4/ND	144.9	1.3	18	62.8	84	286/ul	Culture	Pus	N.caviae	ND
13	28/ND	62.4	0.4	27.7	122.9	82	467/ul	Culture	Pus, sputum, pleural effusion	ND	ND
14	34/ND	90.8	0.4	22.3	136.1	66	817/ul	Culture	Pus	N.brasiliensis	ND
15	11.1/ND	152.1	1.1	33	50.4	128	ND	Culture	Pleural effusion	ND	ND
16	17.4/ND	202.1	0.8	30.2	61	122	ND	Culture	Pus, lung fine needle aspiration biopsy	ND	ND
17	7.1/ND	160.3	0.2	25	67.2	109	126/ul	Culture	Pus	ND	ND
18	16.5/ND	140	1.4	24	83.1	128	177/ul	Culture	Pleural effusion	ND	ND
19	18.2/ND	18	0.2	26.5	55.7	107	661/ul	Culture	Pus	ND	ND
20	6.0/ND	187.4	1.6	14.5	98.1	109	143/ul	Culture	Sputum	ND	ND
21	14.5/ND	101.8	18.2	21.9	129.1	123	481/ul	Culture	Pus, sputum	N.farcinica	ND
22	19.7/85	8.4	0.9	20.2	139.3	ND	ND	Culture	Pus	ND	ND
23	15.0/86.8	26.6	0.3	24.3	73.4	ND	ND	Culture	Pus	ND	ND
24	18.2/95.9	116	0.9	24.2	71.6	ND	ND	Culture	Pus	ND	ND
25	10.6/91.1	306	0.5	24.5	74.3	ND	ND	Culture	Pus	ND	ND
26	16.5/93.4	10.4	0.4	24.2	46.6	ND	ND	Culture	Pus	ND	ND
27	28.9/97.8	ND	ND	25.5	188	106	ND	ND	Lung biopsy tissue, pus	ND	ND

ND, not detected; WBC, white blood cell; CRP, C-reactive protein; PCT, procalcitonin; Alb, albumin; SCr, Serum creatinine; BALF, Bronchoalveolar lavage fluid; AST, antimicrobial susceptibility testing; 16S rRNA, 16S ribosomal RNA gene sequencing; MALDI-TOF MS, matrix-assisted laser desorption ionization time-of-flight mass spectrometry.

### Epidemiographical Data of NS Patients Infected With *Nocardia*

**Figure 3:**
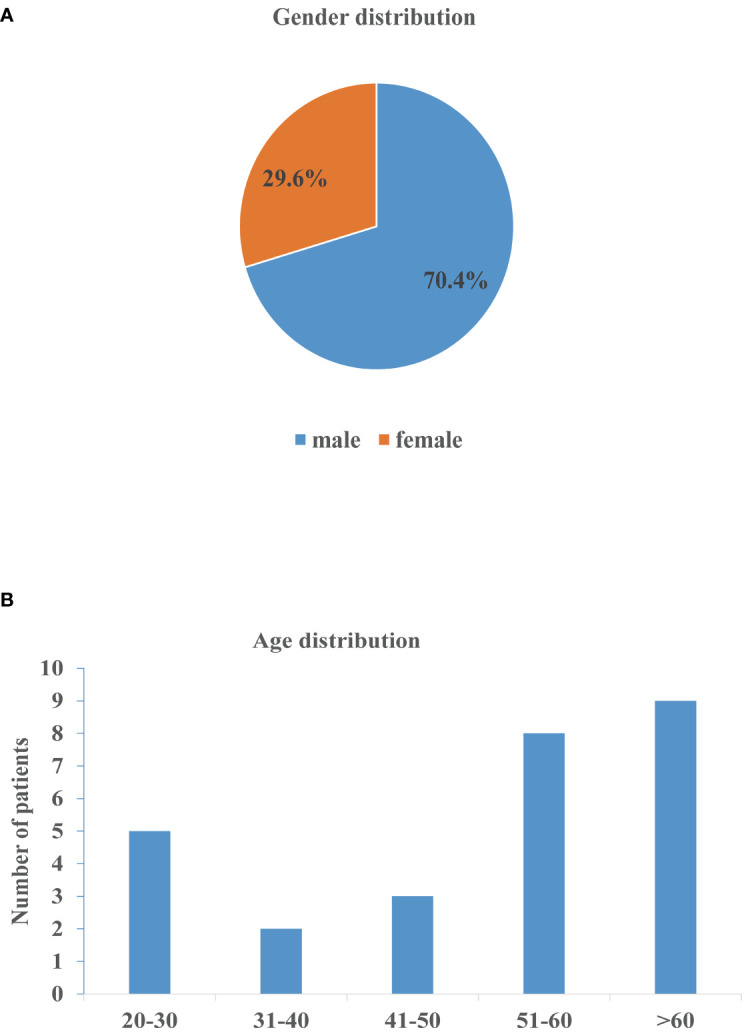
Gender and age distribution of nocardiosis with nephrotic syndrome. **(A)** Gender distribution of patients. **(B)** Age distribution of patients.

The demographic characteristics of the patients in the articles reviewed included 19 males (70.4%) and 8 females (29.6%) (**Figure 3A**). A slight increase in the numbers of patients was observed in the age group 51-60 and > 60 years of age (**Figure 3B**). Concomitant diseases observed were chronic obstructive pulmonary disease (COPD) in 2 patients (case 2 and 3), HIV infection in 1 patient (case 8), chronic hepatitis B virus (HBV) infection in 2 patients (case 5 and 23), and underlying diabetes mellitus in 3 patients (case 2, 17 and 24) (**Table 1**). Furthermore, 10 cases were diagnosed as steroid diabetes after glucocorticoid treatment.

### Characteristics and Treatment of NS

The NS type was confirmed by renal needle biopsy. The pathological examination confirmed 13 cases of membranous nephropathy, 3 cases of lupus nephritis, 2 cases of focal segmental glomerulosclerosis, 2 cases of minimal change nephrosis, 1 case of mesangial proliferative glomerulonephritis, and 1 case of immunoglobulin A nephropathy (**Table 1**). All patients had received glucocorticoid or corticosteroid therapy (case 7) for NS with mean doses of 30.9 ± 13.7 mg/d when infected (case 6 and 27 without doses of glucocorticoid). Combination treatments of glucocorticoid and immunosuppressor were administrated in 17 patients. The average time between hormone therapy and *Nocardia* infection was 8.5 ± 9.7 months. Nineteen patients developed nocardiosis within 6 months after glucocorticoid or corticosteroid therapy. Case 7 was diagnosed as disseminated nocardiosis within 2 months of corticosteroid therapy.

### Clinical and Radiographic Manifestations of Nocardiosis

Serum albumin levels were reported in 23/27 cases, with all 23 patients having hypoalbuminemia; 68% (15/22) of the patients had increased serum creatinine levels (**Table 2**); and 13 cases had anemia, with the average hemoglobin level at 103.9 ± 18.1 g/L. The elevated WBC counts were noted in 24 out of the 27 cases (88.9%), and the average level was 17.0 ± 6.5×10^9^/L. The neutrophils percentage was reported in 15/26 cases, and all had elevated neutrophil percentages. Case 6 had a normal WBC count but with an elevated neutrophil percentage. The CRP levels were reported in 24/27 cases; all but 1 (95.8%) had elevated CRP levels. Serum PCT levels were reported in 21/27 cases, and the PCT levels were ≥0.5 ng/mL in 13 patients; however, the PCT level was significantly elevated in only one patient (case 21). The average CRP and PCT were 101.3 ± 73.8 mg/L and 1.7 ± 3.7 ng/mL, respectively. The CD4^+^ T-lymphocyte counts were tested in 12 patients, and 9 of them had decreased CD4^+^ counts.

In our retrospective study, pulmonary infection was the most common manifestation in NS patients, with 85.2% (23/27) of patients affected (**Table 1**). The disseminated infection occurred in 77.8% (21/27) patients, 29.6% (8/27) patients suffered from multiorgan dissemination (>2 organ), and 5 cases had evidence of central nervous system (CNS) infection, and only 1 patient developed *Nocardia* bacteremia, a rare occurrence (case 4). Other infection sites of nocardiosis were skin (12/27), pleural cavity (3/27), hip (3/27), eye (2/27), and neck (2/27).

The main respiratory symptoms included fever (74.1%, 20/27), cough (48.1%, 13/27), expectoration (44.4%, 12/27), chest pain (29.6%, 8/27), dyspnea (11.1%, 3/27), chest tightness (7.4%, 2/27), and chill (7.4%, 2/27) (**Table 1**). Multiple pulmonary symptoms manifested in one patient. Subcutaneous or skin abscesses were observed in 18 patients. Subcutaneous abscesses were observed in 7 patients as the first symptoms.

Chest CT images revealed that pulmonary nodules/mass (48.1%, 13/27), consolidation (37%, 10/27), and pleural effusion (37%, 10/27) were the most common manifestations. Other pulmonary radiological findings included pleural thickening (18.5%, 5/27), cavities (18.5%, 5/27), bronchiectasis (18.5%, 5/27), pericardial effusion (18.5%, 5/27), pulmonary abscesses (14.8%, 4/27), emphysema (7.4%, 2/27), pulmonary bullae (7.4%, 2/27), and ground-glass opacity (3.7%, 1/27). Magnetic resonance imaging (MRI) and CT showed 5 patients with cerebral abscesses; but 2 had no CNS symptoms.

The etiological diagnosis of nocardiosis was established by Gram staining, modified acid-fast staining, microbial culture, gene sequencing, and matrix assisted laser desorption ionization-time of flight mass spectrometry (MALDI-TOF MS). The most common specimen types for microbiological identification were pus (66.7%, 18/27) and sputum (33.3%, 9/27). Six different *Nocardia* species, including *N.cyriacigeorgica, N.brasiliensis, N.farcinica, N.otitidiscaviarum, N.asteroides* and *N.caviae*, were identified in 13 cases, 6 cases by 16S rRNA gene sequencing, 6 cases by culture and biochemical tests, and 1 case by MALDI-TOF MS. AST was performed in 8 cases.

### Nocardiosis Treatment and Patient Prognosis

All 27 patients received antibiotic treatment once they were diagnosed, of which 25 patients were treated with TMP-SMX monotherapy (18.5%, 5/27) or TMP-SMX-based multidrug therapy (74.1%, 20/27). Only 2 patients (case 12 and 14) received biapenem monotherapy. Thirteen patients having subcutaneous abscesses or pericardial fluid received surgical drainage in addition to antibiotic therapy. During follow-up, except for case 27 (which lacked the treatment duration), the antibiotic therapy duration in other cases lasted from 3 months to 1 year. In terms of prognosis, all patients improved after antibiotic therapy and abscess drainage, but 2 patients (case 2 and 24) were lost to follow-up. In case 24, the abscess in the right middle finger diminished after 3-day antibiotic treatment, but he was lost to follow-up. Case 2 refused further treatment despite clinical improvements after 21 days of hospitalization.

## Discussion

Nocardiosis is a rare infectious disease caused by the filamentous Gram-positive bacteria *Nocardia* spp. present in the environment. Nocardiosis most frequently presents with pulmonary, cutaneous, and subcutaneous infections, leading to invasive and potentially disseminated infections in severe cases.

Previous research (Martinez-Barricarte, 2020) showed that *Nocardia* infection had gender biases, possibly due to differences in distinct lifestyles and professions of man and women; nocardiosis mainly occurred in the age groups 31-40 and 51-60 years. The present study observed males as a more high-risk infection category than females with an incidence of 2:1, similar to the observations in immunocompromised individuals (Beaman et al., 1976). We also found that there was no age predilection for infection in NS patients in the age group 31-40 years, however, those over 50 years were impacted.

Long-term glucocorticoids and immunosuppressive therapy, which is proposed to be the leading risk factor for nocardiosis, can inhibit host cell-mediated immunity against infection (Ercibengoa et al., 2020). Patients with deficient cell-mediated immunity predispose to nocardiosis (Oh et al., 1988; Penkert et al., 2016; Sheikh-Taha and Corman, 2017). Previous studies confirmed that impaired renal function might compromise normal immune function, and the elevated serum creatinine levels in NS patients was an independent risk factor for pulmonary infection (Xu et al., 2017; Wang et al., 2019). Other predisposing factors, including organ transplantation, diabetes mellitus, HIV infection, and COPD (Saubolle & Sussland, 2003; Filice, 2005; Rosen et al., 2015; Coussement et al., 2017; Haussaire et al., 2017), could further destroy the immune system of patients and increase the risk of *Nocardia* infection. In our retrospective study, the treatment of glucocorticoids and (or) immunosuppressant and elevated serum creatinine indicated impaired immune function, and the patients were predisposed to infection. Additionally, 14 patients (51.9%) had two or three other risk factors, suggesting that these patients were more likely to be at a higher risk of *Nocardia* infection. Case 3 developed nocardiosis 1 month after the low-dose (15 mg/d) prednisone therapy, suggesting that opportunistic infection by rare pathogens may also occur during low-dose glucocorticoid maintenance therapy.

The lung is the most common site of infection because inhalation is the primary entry route for *Nocardia* spp., accounting for 62-86% of infection. The frequently involved organs include the CNS and skin, and concerns 2%-42.9% and 8%-31% of patients with *Nocardia* infection, respectively (Minero et al., 2009; Coussement et al., 2016; Haussaire et al., 2017; Yagishita et al., 2021). In our study, the NS patients did not have any trauma history, so it was assumed that the pathogen might infect patients through the respiratory tracts and then disseminate into the skin. Our data indicated that cutaneous nocardiosis was more common than CNS nocardiosis in NS patients. However, Yagishita et al. (Yagishita et al., 2021) reported that brain infection (35.7%, 5/14) was more than skin/cutaneous infection (28.6%, 4/14) in patients with connective tissue diseases. The possible explanation for this difference in the infected organs needs further studies. Interestingly, compared with other immunosuppressed patients, such as Cushing’s Syndrome patients [77.8% (21/27) *vs* 38.9% (7/18), *P*=0.013] (Zhang et al., 2021) and solid organ transplant recipients [77.8% (21/27) *vs* 42.7% (50/117), *P*=0.001] (Coussement et al., 2016), NS patients had a higher rate of disseminated infection. A possible explanation could be the presence of disseminated infections already even before the appearance of apparent symptoms due to its low virulence, slow growth, and long infection duration. Therefore, if cutaneous lesions were identified, disseminated *Nocardia* infection should be considered. Virulent *Nocardia* gets rapidly cleared from the blood (Beaman and Beaman, 1994), making its spread detection through the bloodstream difficult. Our study showed that only 3.7% (1/27) of the patients experiencing *Nocardia* bacteremia by the automated blood culture system were detected. *Nocardia* bacteremia might be underestimated due to its slow growth, low grade or intermittent bacteremia, bacteremia only at very initial moments of the infections. A study of solid organ transplant recipients with CNS nocardiosis showed that 43.3% of cases had no CNS manifestations, and unrecognized CNS involvement might potentially lead to the treatment failure (Coussement et al., 2016). We found that 3 CNS nocardiosis patients with cerebral abscesses had no neurological symptoms, highlighting the importance of performing routine contrast-enhanced brain imaging for all patients with demonstrated or suspected nocardiosis.

The main clinical characteristics and symptoms of pulmonary *Nocardia* infection in NS patients include fever, cough, expectoration, chest pain, dyspnea, chest tightness, and chill, indicating that it is non-specific and difficult to distinguish from other bacterial infections. Cutaneous infection in NS patients manifest as ulcers, abscesses and nodules, and can spread to lymph nodes. If the infection spreads to the CNS, the symptoms include weakness, ataxia, and sudden obnubilation. Previous research demonstrated that primary skin and subcutaneous lesions were rare manifestations of *Nocardia* infection (Shimizu et al., 1998). In NS patients, both respiratory symptoms and multiple subcutaneous indurations or abscesses could be the first symptoms. Some patients had no pulmonary symptoms, while some experienced respiratory distress. Case 7 was shifted to ICU and intubated because of respiratory distress. Case 6 had inflammatory nodules in the lung without fever or any pulmonary symptoms; his infection manifested as the primary subcutaneous abscess. Therefore, a physician should consider the *Nocardia* infection when the skin and subcutaneous lesions are identified in NS patients receiving immunosuppressive therapy.

In our study, nodule/mass and consolidations were the major radiologic manifestations in NS patients with *Nocardia* infections, consistent with the previous single-center review (Guo et al., 2020; Han et al., 2020). A retrospective study of Cushing’s syndrome with nocardiosis reported that cavitary lesions and nodules were the most common radiological findings (Zhang et al., 2021), indicating that the main radiologic manifestations may have slight differences in different diseases. Han et al. (Han et al., 2020) proposed that pulmonary abscesses were important signs of nocardiosis in glomerular disease patients, whereas only 4 patients (case 2, 4, 25, 27) presented with pulmonary abscesses in our review. Therefore, further studies with more cases are needed to assess if pulmonary abscesses were important characteristics in NS patients and the radiological differences between NS patients and other nocardiosis.

Procalcitonin is a well-known inflammatory biomarker for early detection of bacterial infections, with its levels increasing from systemic inflammatory response syndrome (0.6-2 ng/ml) to severe sepsis (2-10 ng/ml) and septic shock (>10 ng/ml) (Markanday, 2015). The antibiotics administration is usually encouraged at the PCT levels ≥0.5 ng/mL (Bouadma et al., 2010). Nevertheless, previous research demonstrated inadequate sensitivity of PCT for the early diagnosis of Gram-positive bacterial infections (Koizumi et al., 2020). In our study, normal or slightly elevated PCT levels in most nocardiosis patients could be because *Nocardia* is Gram-positive bacteria and most *Nocardia* species have low virulence. Further research on the role of PCT levels in the early diagnosis of *Nocardia* infections requires more clinical data.

Non-specific and diverse clinical characteristics and radiological manifestations of nocardiosis make its diagnosis difficult as these findings are similar to fungal, mycobacterial, and other bacterial infections. Therefore, microbiological identification plays a vital role in the diagnosis of nocardiosis. Microscopic examination, performed mainly by Gram-staining and modified acid-fast staining, can provide an early suspicion of nocardiosis. If *Nocardia* infection is suspected, prolonged incubation and the use of specific media, such as mycobacterial medium, buffered charcoal-yeast extract (BCYE), and fungal medium, are recommended (Saubolle and Sussland, 2003; Brown-Elliott et al., 2006). Most cases in our retrospective study were diagnosed through the microbial culture of clinical specimens. Case 3 proved that mycobacterial medium and digestion with 2% KOH before inoculation were useful for inhibiting the oropharyngeal flora growth to improve the separation rate. In addition to cultures, *Nocardia* may be identified by polymerase chain reaction (PCR)-based rapid molecular techniques on clinical samples with high sensitivity but relatively lower specificity (Couble et al., 2005; Rouzaud et al., 2018). Therefore, positive PCR results of respiratory samples might be an indication of colonization and not of infection (Brown-Elliott et al., 2006; Coussement et al., 2019). Reliable species identification can predict antimicrobial susceptibility and guide the initial antimicrobial therapy before obtaining antibiotic susceptibility patterns (Wallace et al., 1991; McTaggart et al., 2015; Body et al., 2018; Durand et al., 2020). The gold standard for identifying *Nocardia* species is molecular biology, such as PCR-based assay and 16S rRNA gene sequencing (Conville et al., 2012; Valdezate et al., 2017; Martinez-Barricarte, 2020). In addition, MALDI-TOF MS is being used increasingly to identify *Nocardia* species (Girard et al., 2017; Body et al., 2018). In case 7, initial multidrug therapy (amikacin, meropenem, and cotrimoxazole) was effective; however, meropenem was then modified to linezolid as the species identified, *N.otitidiscaviarum*, was less susceptible to beta-lactam antibiotics. Thus, accurate species identification will facilitate the selection of initial antimicrobial therapy.

To date, there is no consensus for optimal management of nocardiosis. Carbapenem monotherapy has been found effective for nocardiosis (Ameen et al., 2010); 2 cases (case 12 and 14) in our study confirmed the effectiveness of carbapenem monotherapy of disseminated nocardia infection in NS patients. Sulphonamides, most commonly TMP-SMX, have been the mainstay treatment for *Nocardia* infections since the 1950s (Wilson, 2012). Additionally, combination drug therapy (two or three drugs) had been suggested, especially for patients with serious or disseminated infections (Liu et al., 2017). The companion regimens in TMP-SMX-based multidrug therapy include third-generation cephalosporins (ceftriaxone, cefotaxime), carbapenems (imipenem, meropenem, ertapenem), amikacin, and linezolid. Linezolid may be used as monotherapy for nocardiosis treatment because of its excellent coverage against all pathogenic *Nocardia* species (Shen et al., 2011; Davidson et al., 2020). If antimicrobial treatment fails to control the infection, we should take therapeutic modifications into account. In case 4, the patient was administered with oral TMP-SMX in combination with intravenous imipenem/cilastatin because the initial antibiotic treatment was ineffective. In case 15, antibiotic treatment was switched to intravenous biapenem and linezolid for 2 weeks because of aggravated pulmonary inflammation and multiple cerebral abscesses after initial TMP-SMX monotherapy. An antibiotic duration of 3-12 months is recommended depending on the therapeutic response, infection site, and immune status of patients (Wallace et al., 1982; Hui et al., 2003; Wilson, 2012). It is seemed that the mortality rate of nocardiosis in NS patients is far lower than that in other immunocompromised patients of 40-66.7% (Xu et al., 2016; Williams et al., 2020; Zhang et al., 2021). One reason could be no co-infections with other pathogens, associated with increased mortality in nocardiosis (Huang et al., 2019). Another reason could be the effective treatment of CNS infection. The mortality rate of CNS infection in SLE (Systemic Lupus Erythematosus) and Cushing’s Syndrome was more than 50% (Mc-Nab et al., 2000; Zhang et al., 2021). Multidrug therapy is preferable, and neurosurgical drainage should be especially considered for patients with large brain abscesses or not responding to antimicrobial therapy, or if a co-infection is suspected (Anagnostou et al., 2014; Rafiei et al., 2016; Restrepo et al., 2019). In our retrospective study, CNS infection occurred in 5 of 27 patients; 4 of them received TMP-SMX-based multidrug therapy (two or three drugs). All patients with CNS nocardia infection recovered without surgical drainage and did not relapse during the follow-up period, indicating that multidrug therapy was effective. Martinez-Barricarte (Martinez-Barricarte, 2020) demonstrated that antibiotic treatment was used in combination with debridement or drainage of affected area in the lymph nodes, CNS, lungs, or extremities in 18.3% of cases. In our study, antibiotic therapy was combined with surgical drainage in nearly 50% of cases with subcutaneous abscesses or pericardial fluid, and these results demonstrated the combination strategy might play an important role in patients’ therapeutic outcomes.

Our retrospective study has a few limitations. First, despite a multicenter retrospective study, the number and area of reported nocardiosis cases with NS are limited. Second, some laboratory and clinical testing, such as brain imaging, AST, species identification, were not performed in some cases, which may generate some bias when analyzing clinical data.

## Conclusions

NS patients who received low-to-high doses of immuno-suppressive therapies within 6 months are at high risk of *Nocardia* infection. Nocardiosis in NS patients had a high rate of disseminated and cutaneous infection but a low mortality rate. Physicians should be alert about *Nocardia* infection in NS patients presenting subcutaneous abscesses or nodules and inform the microbiology laboratory of the suspected nocardiosis. As demonstrated in our case reports, the improvement of laboratory diagnostic methods, including careful microscopic examination, prolonged incubation, use of appropriate media, accurate species identification, and antimicrobial susceptibility testing, should be highlighted for timely diagnosis and correct treatment of nocardiosis. A combination of accurate antibiotic therapy and surgical drainage might be essential for the successful treatment and prognosis of patients.

## Data Availability Statement

The original contributions presented in the study are included in the article/**Supplementary Material**. Further inquiries can be directed to the first author.

## Ethics Statement

The studies involving human participants were reviewed and approved by The Ethics Committee of 980th Hospital of the PLA Joint Logistical Support Force (No.2021-KY-121). The patients/participants provided their written informed consent to participate in this study. Written informed consent was obtained from the individual(s) for the publication of any potentially identifiable images or data included in this article.

## Author Contributions

All authors contributed to this work. All authors have read and agreed to the published version of the manuscript. YC and JG designed the study. T-yW, H-lY, WL, and Z-xH collected the data. J-pS, JC, J-yG, and F-kW interpreted the data. YC wrote the first draft of the paper. YC and JG reviewed and approved the final report.

## Funding

This research was funded by the National Natural Science Foundation of China (No.31200142).

## Conflict of Interest

The authors declare that the research was conducted in the absence of any commercial or financial relationships that could be construed as a potential conflict of interest.

## Publisher’s Note

All claims expressed in this article are solely those of the authors and do not necessarily represent those of their affiliated organizations, or those of the publisher, the editors and the reviewers. Any product that may be evaluated in this article, or claim that may be made by its manufacturer, is not guaranteed or endorsed by the publisher.

## Supplementary Material

Supplementary Figure S1 Bronchoscopic images and magnetic resonance imaging of brain in case 1. **(A)** Bronchoscopic image at the left lower lobe. **(B)** Bronchoscopic image at the right middle and lower lobe. **(C)** Magnetic resonance imaging of brain.

Supplementary Figure S2 16S rRNA gene sequencing of *Nocardia* spp. isolated from BALF in case 1. **(A)** Gene sequencing of *N.cyriacigeorgica* identified from BALF. **(B)** Sequence blast description.

Supplementary Figure S3 CT scanning and microbiological identification of case 2. **(A)** Gram staining of sputum showing Gram-positive, filamentous branching bacilli (magnification, ×100). **(B)** Modified acid-fast staining of sputum showing filamentous, weakly acid-fast branching bacilli (magnification,×100). **(C)** Colonies of *N.brasiliensis* cultured on blood agar for 72h. **(D)** Gram staining showing the Gram-positive, filamentous branching and beaded structure of *N.brasiliensis* (magnification,×100). **(E)** Modified acid-fast staining showing filamentous, weakly acid-fast branching bacilli (magnification,×100). **(F)** Chest CT showing multiple cavities in the right upper lobe and bilateral pleural effusion. **(G)** Chest CT showing the size of abscesses decreased gradually following treatment.

Supplementary Figure S4 16S rRNA gene sequencing of *Nocardia* spp. isolated from sputum in case 2. (A) Gene sequencing of *N.brasiliensis* identified from sputum. (B) Sequence blast description.

Supplementary Figure S5 Treatment flow diagram of case 2. TMP-SMX: trimethoprim-sulfamethoxazole.

Supplementary Figure S6 CT scanning, ultrasonography and microbiological identification of case 3. **(A)** Gram staining of sputum showing Gram-positive, filamentous branching bacilli (magnification, ×100). **(B)** Modified acid-fast staining of sputum showing filamentous, weakly acid-fast branching bacilli (magnification,×100). **(C)** Colonies of *N.farcinica* cultured on blood agar for 72h. **(D)** Colonies of *N.farcinica* cultured on mycobacterial medium (Lowenstein-Jensen medium) for 72h. **(E)** Gram staining showing the Gram-positive, filamentous branching and beaded structure of *N.farcinica* (magnification,×100). **(F)** Modified acid-fast staining showing filamentous, weakly acid-fast branching bacilli (magnification,×100). **(G)** Chest CT showing cavities in the right upper lobe. **(H)** Chest CT showing nodules in the left lung. **(I)** Chest CT showing nodules in the right lung. **(J)** USG of abscess showed thickened subcutaneous soft tissue and the internal echo was not uniform.

Supplementary Figure S7 16S rRNA gene sequencing of *Nocardia* spp. isolated from sputum and pus in case 3. (A) Gene sequencing of *N.farcinica* identified from sputum. (B) Sequence blast description.

Supplementary Figure S8 Treatment flow diagram of case 3. TMP-SMX: trimethoprim-sulfamethoxazole. T spot-TB: Mycobacterium tuberculosis specific T lymphocyte.
