# Supplementary figures and images for: Nocardia Infection in Nephrotic Syndrome Patients: Three Case Studies and A Systematic Literature Review
Source: Front Cell Infect Microbiol. 2022 Jan 24;11:789754. doi: 10.3389/fcimb.2021.789754 (PMC8819730; doi:10.3389/fcimb.2021.789754)

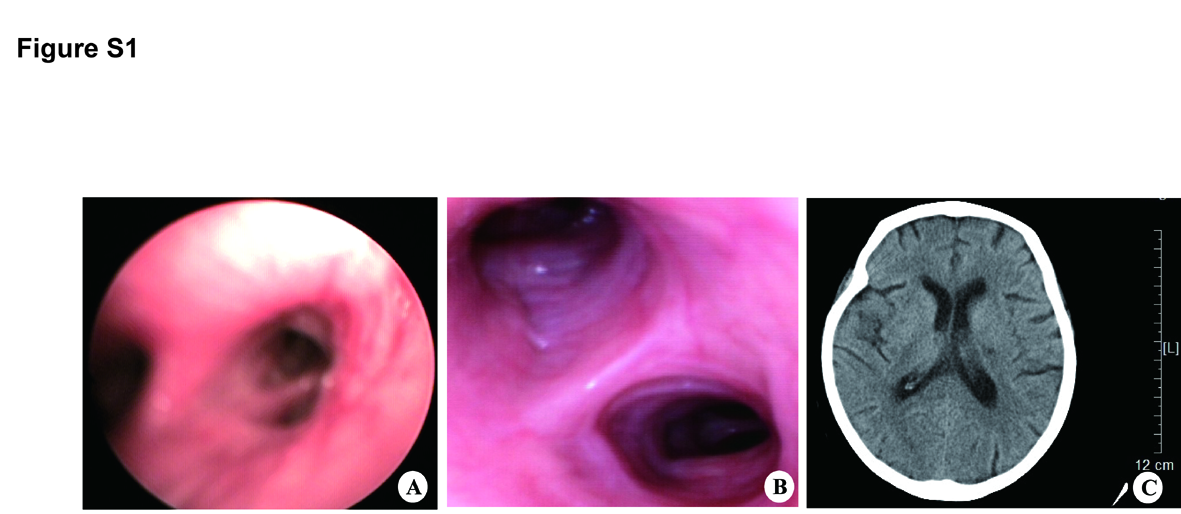

Supplement: Supplementary Figure S1 — Bronchoscopic images and magnetic resonance imaging of brain in case 1. (A) Bronchoscopic image at the left lower lobe. (B) Bronchoscopic image at the right middle and lower lobe. (C) Magnetic resonance imaging of brain. [file Image_1.tif]

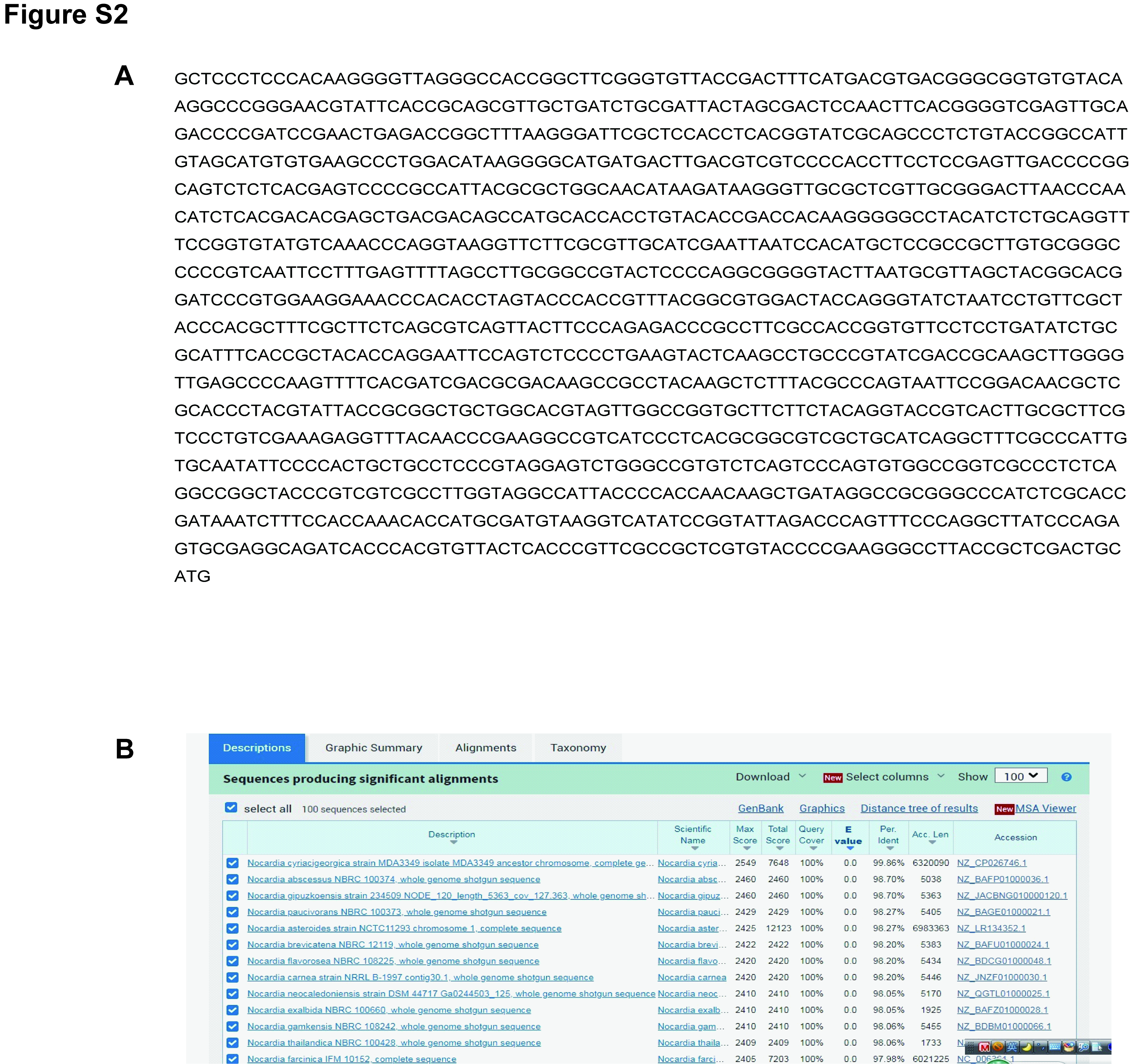

Supplement: Supplementary Figure S2 — 16S rRNA gene sequencing of Nocardia spp. isolated from BALF in case 1. (A) Gene sequencing of N.cyriacigeorgica identified from BALF. (B) Sequence blast description. [file Image_2.tif]

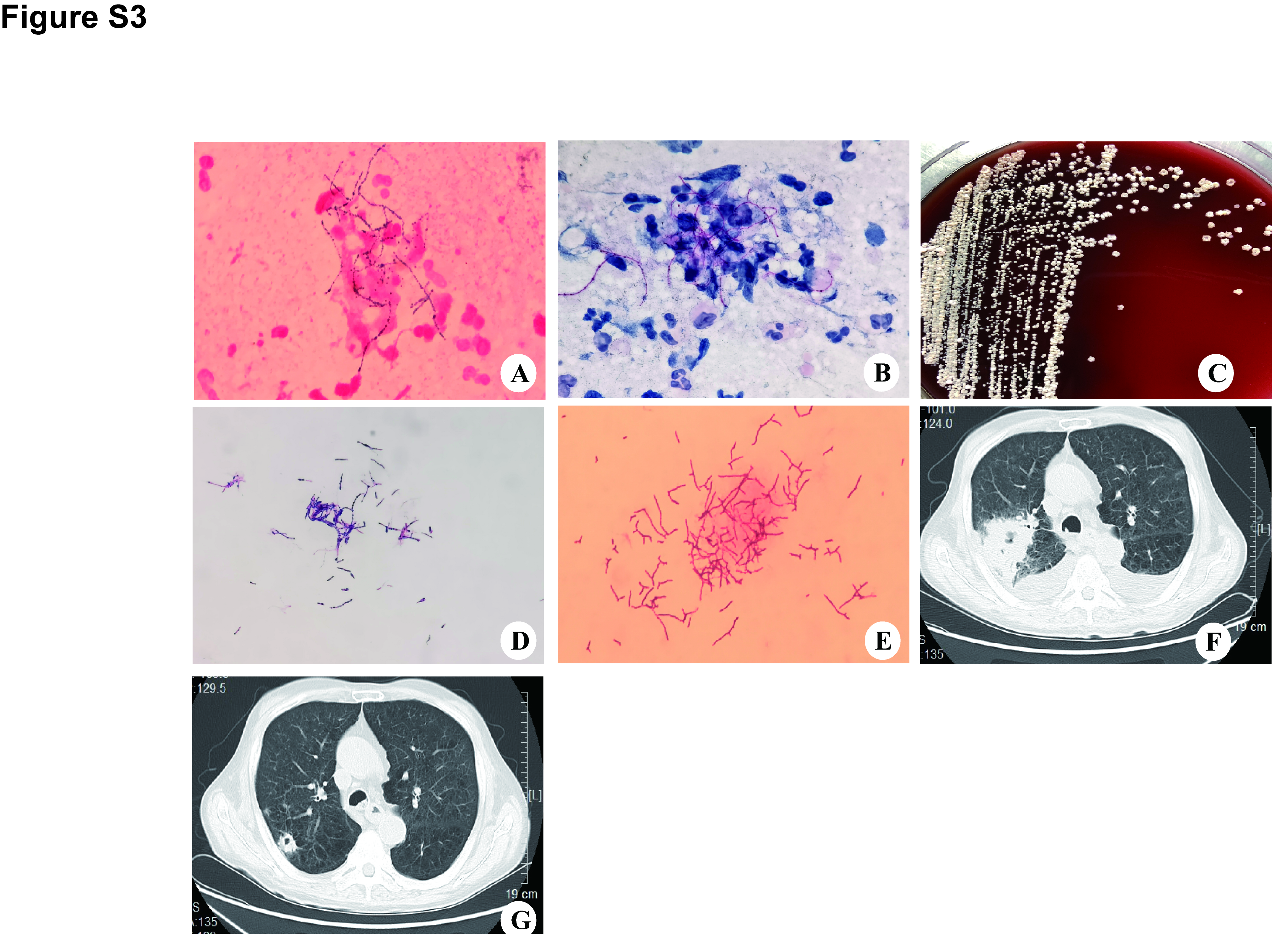

Supplement: Supplementary Figure S3 — CT scanning and microbiological identification of case 2. (A) Gram staining of sputum showing Gram-positive, filamentous branching bacilli (magnification, ×100). (B) Modified acid-fast staining of sputum showing filamentous, weakly acid-fast branching bacilli (magnification,×100). (C) Colonies of N.brasiliensis cultured on blood agar for 72h. (D) Gram staining showing the Gram-positive, filamentous branching and beaded structure of N.brasiliensis (magnification,×100). (E) Modified acid-fast staining showing filamentous, weakly acid-fast branching bacilli (magnification,×100). (F) Chest CT showing multiple cavities in the right upper lobe and bilateral pleural effusion. (G) Chest CT showing the size of abscesses decreased gradually following treatment. [file Image_3.tif]

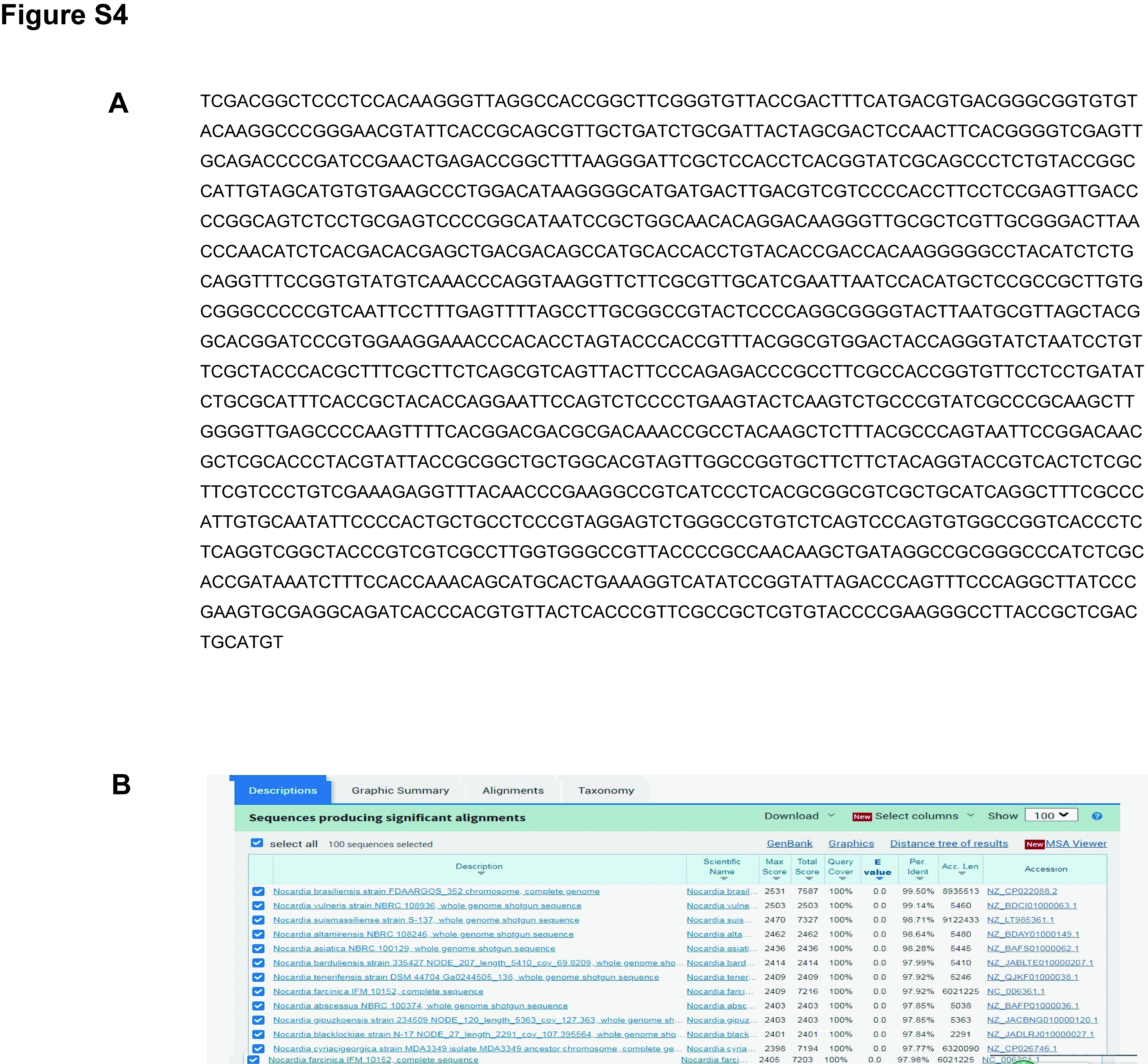

Supplement: Supplementary Figure S4 — 16S rRNA gene sequencing of Nocardia spp. isolated from sputum in case 2. (A) Gene sequencing of N.brasiliensis identified from sputum. (B) Sequence blast description. [file Image_4.tif]

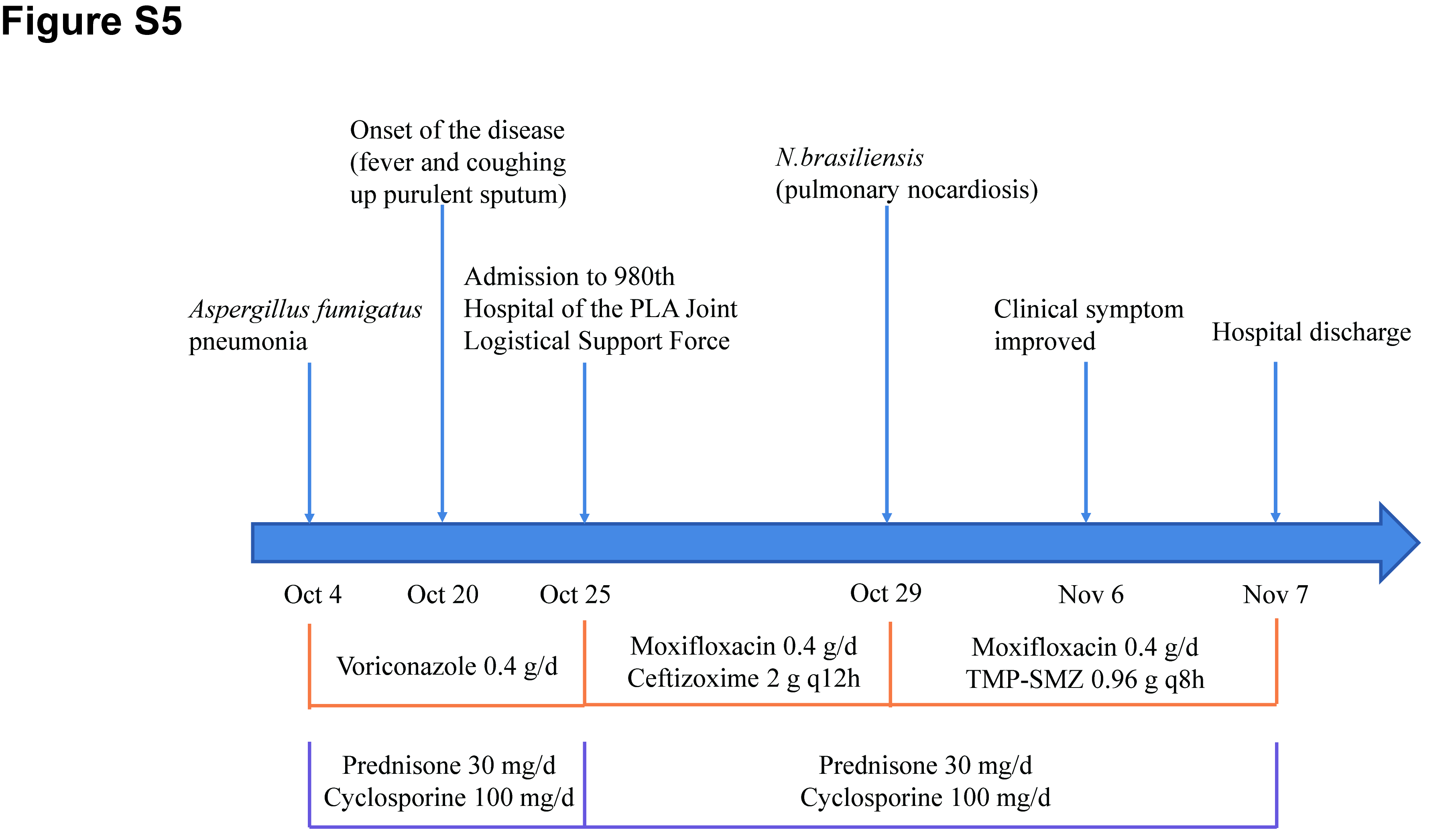

Supplement: Supplementary Figure S5 — Treatment flow diagram of case 2. TMP-SMX: trimethoprim-sulfamethoxazole. [file Image_5.tif]

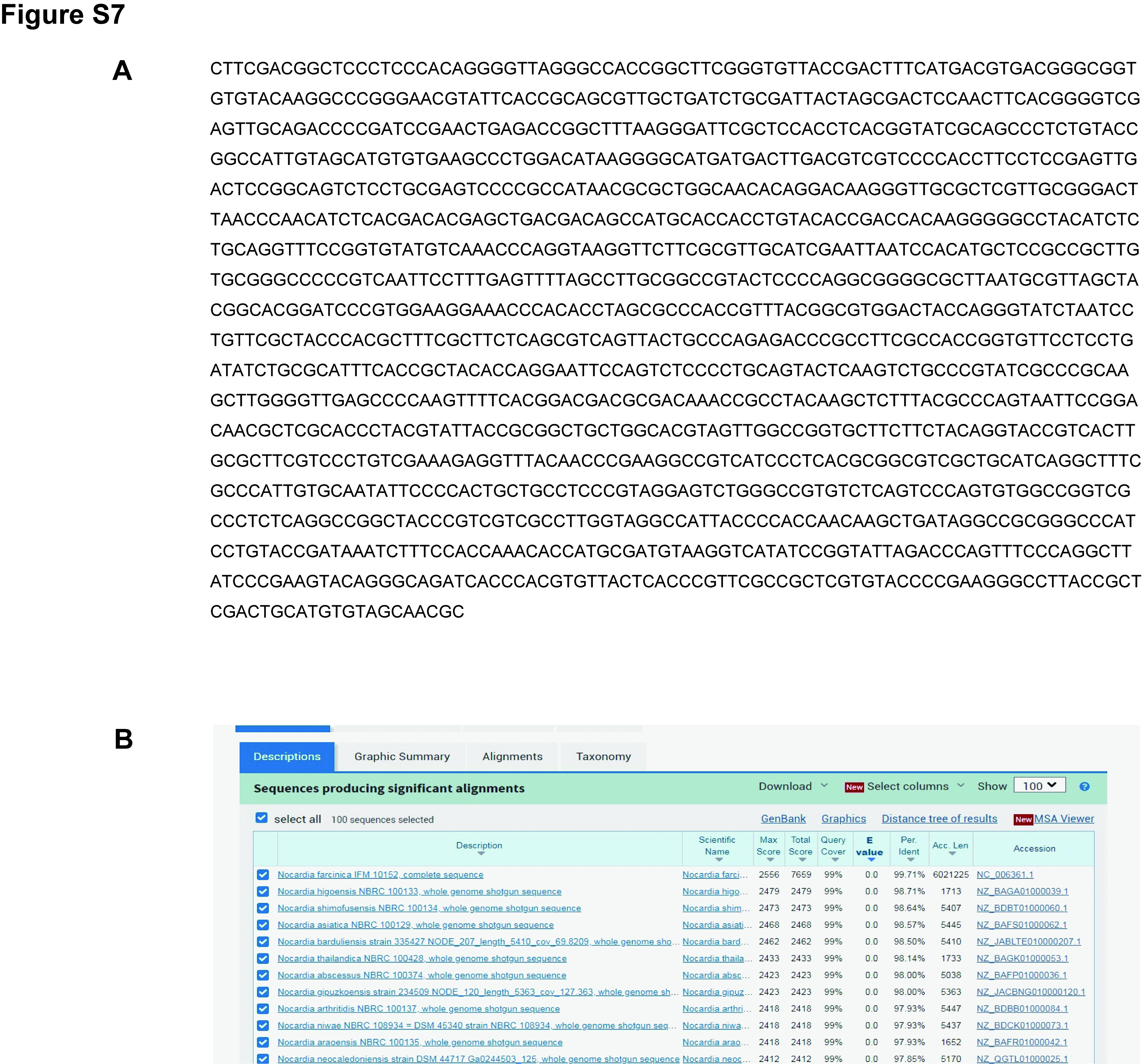

Supplement: Supplementary Figure S6 — CT scanning, ultrasonography and microbiological identification of case 3. (A) Gram staining of sputum showing Gram-positive, filamentous branching bacilli (magnification, ×100). (B) Modified acid-fast staining of sputum showing filamentous, weakly acid-fast branching bacilli (magnification,×100). (C) Colonies of N.farcinica cultured on blood agar for 72h. (D) Colonies of N.farcinica cultured on mycobacterial medium (Lowenstein-Jensen medium) for 72h. (E) Gram staining showing the Gram-positive, filamentous branching and beaded structure of N.farcinica (magnification,×100). (F) Modified acid-fast staining showing filamentous, weakly acid-fast branching bacilli (magnification,×100). (G) Chest CT showing cavities in the right upper lobe. (H) Chest CT showing nodules in the left lung. (I) Chest CT showing nodules in the right lung. (J) USG of abscess showed thickened subcutaneous soft tissue and the internal echo was not uniform. [file Image_6.tif]

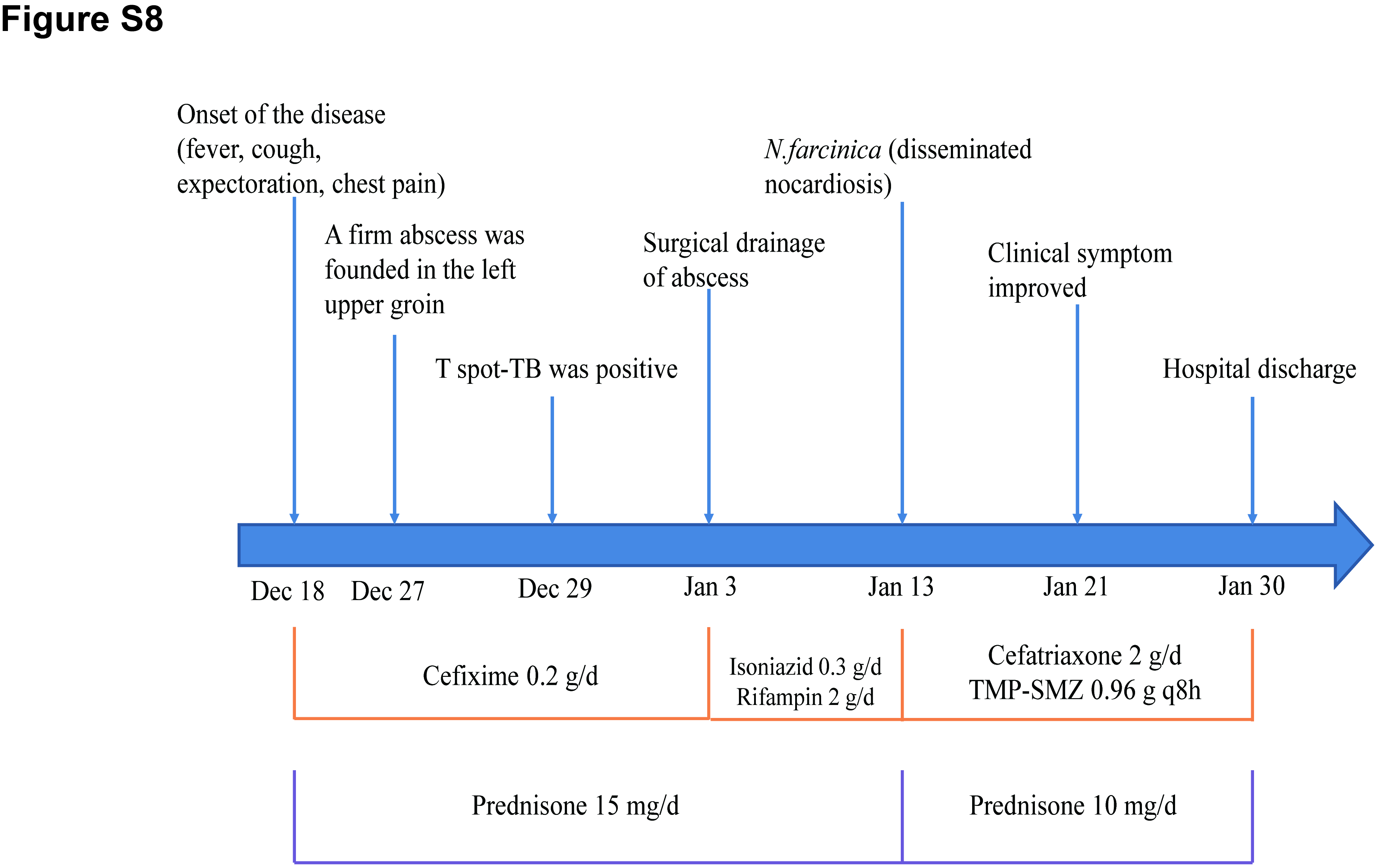

Supplement: Supplementary Figure S7 — 16S rRNA gene sequencing of Nocardia spp. isolated from sputum and pus in case 3. (A) Gene sequencing of N.farcinica identified from sputum. (B) Sequence blast description. [file Image_7.tif]

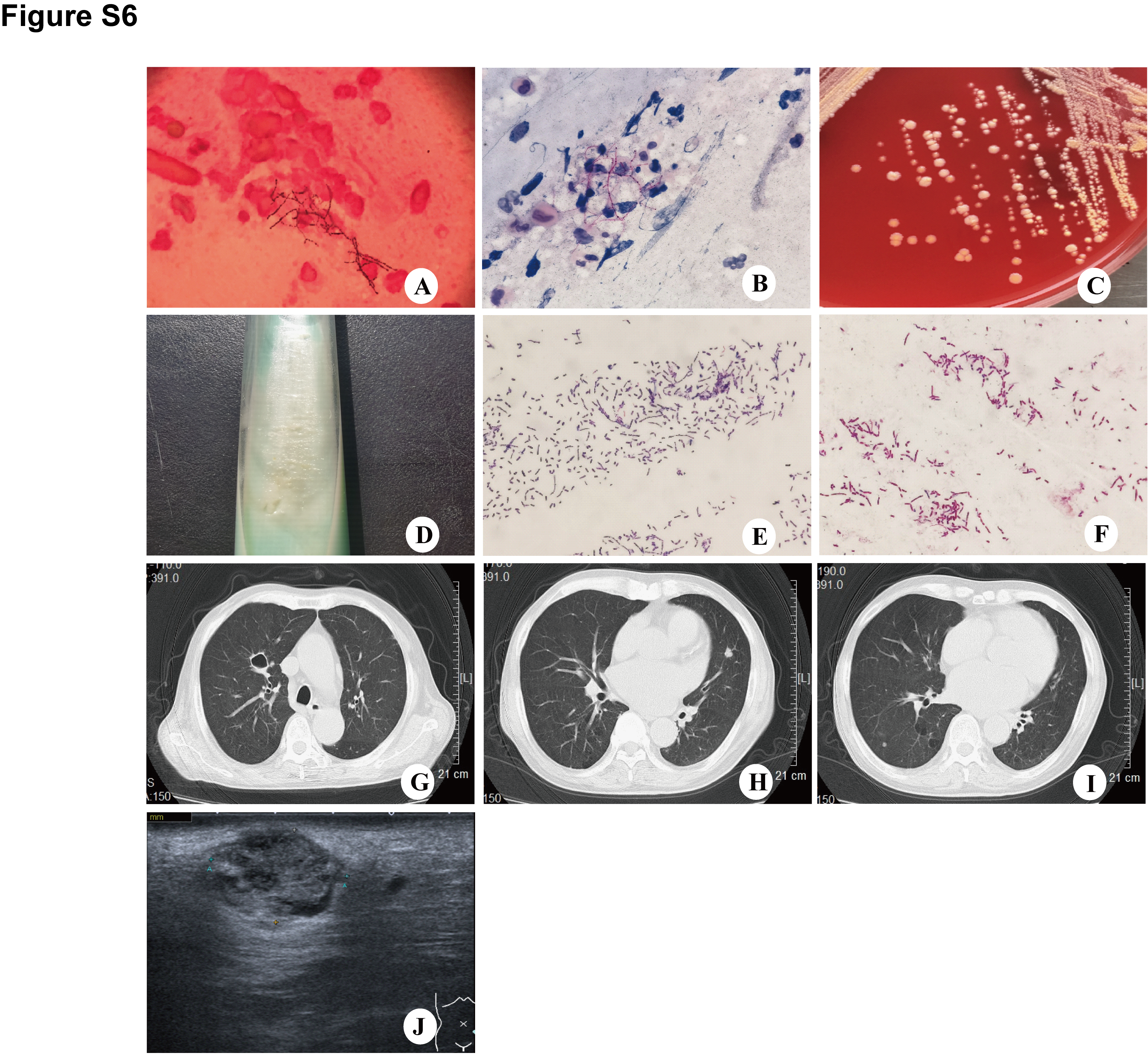

Supplement: Supplementary Figure S8 — Treatment flow diagram of case 3. TMP-SMX: trimethoprim-sulfamethoxazole. T spot-TB: Mycobacterium tuberculosis specific T lymphocyte. [file Image_8.tif]
