# Supplementary material for: Nocardia Infection in Nephrotic Syndrome Patients: Three Case Studies and A Systematic Literature Review
Source: Front Cell Infect Microbiol. 2022 Jan 24;11:789754. doi: 10.3389/fcimb.2021.789754 (PMC8819730; doi:10.3389/fcimb.2021.789754)
Supplement: Supplementary file 9 [file Table_1.docx]

**Table S1 Antimicrobial susceptibility patterns for *Nocardia***

| **Antimicrobial agent** | **Case 1** | | **Case 2** | | **Case 3** | |
| --- | --- | --- | --- | --- | --- | --- |
|  | **MIC(ug/mL)** | **Tentative interpretation** | **MIC(ug/mL)** | **Tentative interpretation** | **MIC(ug/mL)** | **Tentative interpretation** |
| TMP-SMZ | 0.125 | S | 1.5 | S | 0.125 | S |
| Amikacin | 8 | S | 8 | S | 8 | S |
| Ciprofloxacin | 32 | R | 8 | R | 32 | R |
| Ceftriaxone | 0.75 | S | 32 | I | 0.5 | S |
| Cefepime | 16 | I | 8 | S | 3 | S |
| Linezolid | 0.75 | S | 3 | S | 1.5 | S |
| Imipenem | 0.75 | S | 0.75 | S | 0.5 | S |
| Amoxicillin/Clavulanic Acid | 32 | R | 1.5 | S | 48 | R |
| Clarithromycin | 2 | S | 8 | R | 8 | R |
| Tobramycin | 4 | S | 4 | S | 16 | R |
| Minocycline | 1 | S | 1 | S | 1 | S |

TMP-SMX:trimethoprim-sulfamethoxazole, S: susceptible, I: intermediate, R: resistant, MIC: minimum inhibitory concentration.
