# Supplementary material for: Nocardia Infection in Nephrotic Syndrome Patients: Three Case Studies and A Systematic Literature Review
Source: Front Cell Infect Microbiol. 2022 Jan 24;11:789754. doi: 10.3389/fcimb.2021.789754 (PMC8819730; doi:10.3389/fcimb.2021.789754)
Supplement: Supplementary file 10 [file Table_2.docx]

**Table S2 Literature review of publications with cases of Nocardiosis in patients with nephrotic syndrome covering the years 2001-2021**

| **Publication** | **Period of study** | **County** | **Study type** | **Number of patients in study** | **Case number** |
| --- | --- | --- | --- | --- | --- |
| Xu H et al., 2018 | 2018 | China | Case report | 1 | 4 |
| Chen B et al., 2016 | 2015 | China | Case report | 1 | 5 |
| Zhu N et al., 2017 | 2017 | China | Case report | 1 | 6 |
| Sah R et al., 2019 | 2019 | India | Case report | 1 | 7 |
| Zhou L et al., 2014 | 2010 | China | Case report | 1 | 8 |
| Hwang JH et al., 2004 | 2003 | Korea | Case report | 1 | 9 |
| Sirijatuphat R et al., 2013 | 2013 | China | Case report | 1 | 10 |
| Guo J et al., 2020 | 2000-2019 | China | Single-center retrospective review | 11 | 11-21 |
| Han Y et al., 2020 | 2012-2019 | China | Single-center retrospective review | 5 | 22-26 |
| Yang W et al., 2020 | 2020 | USA | Case report | 1 | 27 |
